# A brief guideline for the studies of structure-function relationship of ion channels using AlphaFold3

**DOI:** 10.1016/j.fmre.2025.07.010

**Published:** 2025-07-25

**Authors:** Yichen Ke, Ruijie Gong, Nan Liu, Yaxiong Yang

**Affiliations:** a School of Medicine, Shenzhen Campus of Sun Yat-sen University, Shenzhen, Guangdong 518107, China; b Key Laboratory of Biomechanics and Mechanobiology (Beihang University), Ministry of Education, Key Laboratory of Innovation and Transformation of Advanced Medical Devices, Ministry of Industry and Information Technology, National Medical Innovation Platform for Industry-Education Integration in Advanced Medical Devices (Interdiscipline of Medicine and Engineering), School of Biological Science and Medical Engineering, Beihang University, Beijing 100191, China; c School of Life Sciences, Yunnan University, Kunming, Yunnan 650091, China

**Keywords:** Ion channel, AlphaFold, Structure prediction, Structure-function relationship, P2X receptor, Voltage-gated calcium channel

## Abstract

Ion channels are crucial membrane proteins that regulate ion flux, thus affecting a broad range of physiological processes and disease mechanisms. Traditional approaches, such as X-ray crystallography and cryo-electron microscopy, often face significant challenges in resolving the structure-function relationships of ion channels due to the complex subunit assemblies, transient functional states, and high experimental costs. AlphaFold3 offers a major leap forward by accurately modeling multimeric channels, predicting ligand and ion interactions, and refining subunit interfaces. Building on AlphaFold2, it integrates diffusion-based algorithms and enhanced confidence metrics to explore gating, auxiliary subunits, disease-linked mutations, etc. This review outlines AlphaFold3’s key innovations and provides a step-by-step protocol for its use in predicting ion channel complexes. We discuss essential structural parameters, common software for basic analyses, and complementary techniques including molecular dynamics simulations, molecular docking, functional assays (electrophysiology and ion imaging) and structure biology techniques that extend and validate computational findings. Representative examples, including the P2X receptors and voltage-gated calcium channels, demonstrate AlphaFold3’s ability to clarify channel assembly, gating mechanisms, channelopathies, and therapeutic opportunities. Finally, we address AlphaFold3’s remaining limitations. Despite these hurdles, AlphaFold3 offers a transformative platform that, when integrated with established experimental methods, is poised to significantly advance ion channel research and drug discovery.

## Graphical abstract

**Figure:**
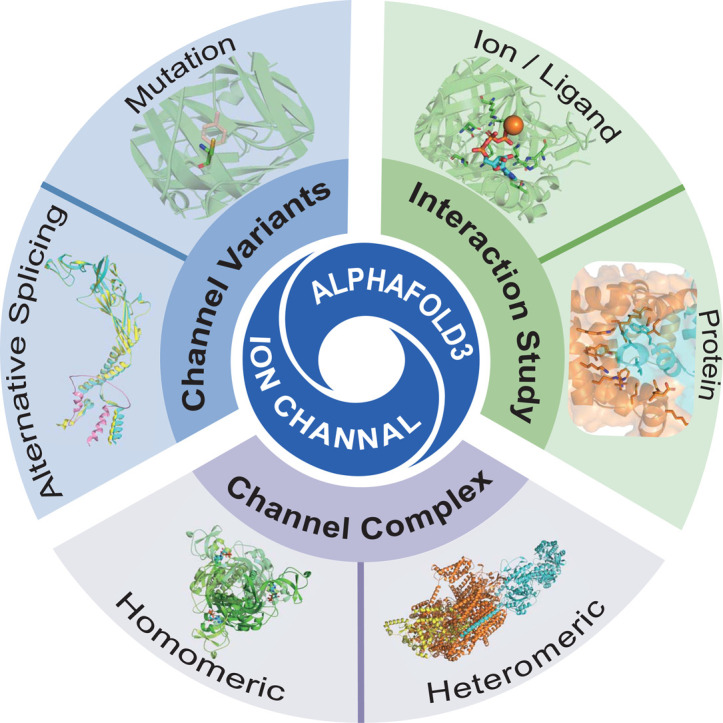

## 1 Introduction to ion channel research

### 1.1 Discovery and significance of ion channels

Ion channels are transmembrane proteins that regulate ion flux across cell membranes, mediating key physiological events such as the genesis and propagation of action potentials, intercellular signaling, and sensory transduction. These processes are critical for vital functions including cardiac rhytshm, neural excitability, and muscle contraction. Accordingly, ion channel dysfunction or channelopathies can cause an array of pathological conditions, including neurological disorders, cardiac arrhythmias, and kidney diseases [1].

The investigation of ion channels traces back to early studies of bioelectricity in the late 18th and early 19th centuries. However, it was not until the mid-20th century that their core functional principles were definitively established. Pioneering research by Hodgkin and Huxley on the squid giant axon led to the ionic hypothesis of the action potential [2], in which they proposed that ion channels function as ion-selective pathways, enabling specific ions to traverse neuronal membranes in response to voltage changes. This seminal work underpins modern electrophysiology and garnered them the Nobel Prize in Physiology or Medicine in 1963. Subsequent milestones in the 1980s ushered in the molecular era of ion channel research, propelled by the advent of molecular cloning. The first cloned ion channel gene, derived from the voltage-gated sodium channel of the electric eel (*Electrophorus electricus*), enabled a detailed molecular characterization of ion channels [3], thereby accelerating knowledge of their architecture and gating.

### 1.2 Classification of ion channels

Ion channels can be broadly classified according to their activation mechanisms into voltage-gated ion channels (VGICs), ligand-gated ion channels (LGICs), mechanically gated ion channels, and additional specialized types.

Voltage-gated ion channels (VGICs) respond to changes in membrane potential and are essential for initiating and propagating action potentials in neurons and muscle cells. VGICs include channels that selectively conduct sodium (voltage-gated Na^+^ channel, Na_V_), potassium (voltage-gated K^+^ channel, K_V_), calcium (voltage-gated Ca^2+^ channel, Ca_V_), and chloride (Cl^-^) ions [4]. Structurally, they commonly consist of either four homologous subunits or four linked, non-identical domains, each harboring six transmembrane helices (S1-S6). The S1-S4 segments constitute the voltage-sensing domain (VSD), where positively charged residues in S4 serve as voltage sensors by undergoing conformational shifts in response to membrane potential changes, thereby regulating channel opening and closure. The S5 and S6 helices form the pore domain, which generally features a selectivity filter for ion discrimination and a lower gate that modulates ion flux.

Ligand-gated ion channels (LGICs), also termed ionotropic receptors, open upon binding of specific extracellular ligands, such as neurotransmitters or environmental molecules (e.g., capsaicin) [5]. These receptors, such as the glutamate receptors, P2X receptors, and transient receptor potential (TRP) channels, mediate rapid synaptic transmission and diverse sensory functions. LGICs typically consist of three to five subunits, each spanning the membrane multiple times, which together form a central ion-conducting pore. Ligand binding triggers conformational changes that govern channel gating [6].

Mechanically gated ion channels are sensitive to mechanical stimuli such as membrane tension, pressure, or stretching forces. They are commonly located in mechanosensory cells, including cochlear hair cells and sensory neurons. Mechanical deformation of the membrane elicits conformational changes in the transmembrane helices or extracellular domains, thereby activating the channel. Piezo channels exemplify this mechanism, given their critical roles in tactile and pressure sensation [7].

In addition to these primary categories, other types of ion channels contribute to critical physiological functions. For instance, leakage channels, such as the sodium leak channel non-selective protein (NALCN) and leak potassium channels, sustain the resting membrane potential through the passive flow of ions [8]. Light-gated ion channels, such as channelrhodopsins, are photoreceptive and open in response to specific wavelengths of light, enabling ion flux across the membrane. They have been instrumental in optogenetics, a technology permitting precise, light-based modulation of neuronal activity [9].

Despite the diversity of their activation and structural features, all ion channels serve a unifying role: transducing external or intracellular cues into ion currents or electrical signals that cells can decode and respond to.

### 1.3 Challenges in studying ion channel structure-function relationships

Advancements in ion channel research have been propelled by various experimental and computational techniques. Among these, the patch-clamp electrophysiology method developed by Neher and Sakmann remains a cornerstone. It enables the direct recording of ionic currents from individual channel or the whole cell expressing channels, thereby providing detailed insights into their gating properties and conductance. This high-resolution approach has been pivotal in elucidating ion channel function at the single-molecule or single-cell level.

Beyond electrophysiology, pharmacological approaches like the use of specific agonists, antagonists, and modulators are widely used to investigate the ion channel functions in native systems, thereby clarifying their physiological and pathological roles. For the study of gating mechanisms, molecular biology and genetic methods like cloning and expression of ion channel genes, combined with site-directed mutagenesis, allow for the investigation of structure-function relationships in recombinant systems. These approaches facilitate understanding of channel gating mechanisms and pathophysiological roles.

Despite their significant contributions, these techniques lack the high-resolution structural data needed to fully elucidate ion channel function. Advanced structural biology techniques, such as X-ray crystallography and cryo-electron microscopy (cryo-EM), have enabled atomic-level resolution of ion channel structures, offering a more direct and compelling approach to investigating structure-function relationships. For instance, the KcsA potassium channel structure clarified the basis of K⁺ selectivity [10], and cryo-EM has revealed more complex channel architectures, such as TRPV1 [11]. Nevertheless, structural studies of ion channels face several challenges. First, ion channels are membrane proteins requiring a lipid environment for stability, making purification and crystallization difficult, particularly given their hydrophobic transmembrane regions and flexible conformations. Second, many ion channels function as components of larger complexes or exist in multiple conformational states, making it challenging to resolve their full assemblies and to stabilize transient states that are critical to understanding gating mechanisms. Third, structural determination using high-resolution methods such as cryo-EM is time-consuming, labor-intensive, and expensive, requiring specialized equipment and expertise that limit the number of laboratories equipped for these analyses. Given the wide variety of ion channels, including various subtypes, splice variants, and species-specific differences, current structural data cover only a fraction of known channels, hindering a comprehensive understanding of their functions.

As a result, the structural information available through experimental methods is insufficient to meet the extensive and in-depth research needs in the field of ion channels. This limitation underscores the importance of computational approaches, such as those offered by AlphaFold3, which can predict high-resolution structures of ion channel complexes.

## 2 Introduction of AlphaFold

The development of the AlphaFold series by DeepMind represents a pivotal milestone in protein structure prediction, culminating in the Nobel Prize in Chemistry in 2024. This groundbreaking method holds the potential to address the longstanding challenge of predicting high-resolution structures, especially in the context of ion channel research.

### 2.1 From AlphaFoldv1 to the breakthrough of AlphaFold2

AlphaFold was not the first method to address protein structure prediction. Before its emergence, many academic teams leveraged the Protein Data Bank (PDB), a repository of experimentally determined protein structures, to develop deep learning-based approaches. Building upon and refining earlier algorithms, the DeepMind team released the first iteration of AlphaFold (AlphaFoldv1). This initial version performed comparably to conventional prediction methods during the 13th Critical Assessment of Structure Prediction (CASP13) competition [12,13], representing a substantial advancement yet still exhibiting certain limitations in accuracy and applicability. In 2020, DeepMind introduced AlphaFold2, a neural network-based computational breakthrough in protein structure modeling. AlphaFold2 employed an end-to-end machine learning pipeline, integrating a novel neural network architecture with training protocols guided by evolutionary, physical, and geometric constraints inherent to protein structures. By harnessing deep learning and multiple sequence alignments (MSA), AlphaFold2 could directly predict the three-dimensional coordinates of every atom in a protein using only its primary amino acid sequence, generating near-atomic-resolution structures without depending on known templates [14,15].

The architecture of AlphaFold2 comprised two major components: the Evoformer module and the structural module. The Evoformer processes multiple sequence alignments (MSA) along with residue-pair representations to extract evolutionary and spatial relationship features. These processed features are then used by the structural module as input to generate an initial set of atomic coordinates. Subsequently, AlphaFold2 employs gradient descent optimization to iteratively refine the atomic coordinates. This optimization utilizes a carefully designed loss function guided by geometric constraints and physicochemical principles, particularly the Frame-Aligned Point Error (FAPE) loss. Specifically, the FAPE loss quantifies structural accuracy by aligning predicted atomic coordinates to local reference frames and calculating positional errors relative to experimentally determined coordinates. This explicit geometric alignment ensures physically plausible structures during the iterative refinement process.

### 2.2 Limitation of AlphaFold2 and introduction of AlphaFold3

Despite AlphaFold2’s strengths in single-protein structure prediction, it faces several notable limitations, especially for applications in ion channel research. Firstly, AlphaFold2 relies heavily on the accuracy of initial structure predictions; inaccuracies in initial inputs may cause gradient descent optimization to become trapped in local minima, reducing overall predictive accuracy. Secondly, AlphaFold2 generally produces a single static structural prediction of proteins in low-energy conformations, restricting its applicability to dynamic processes such as ligand binding or ion channel gating (e.g., open, closed, and intermediate states). It is essential to clarify that the “low-energy conformations” of AlphaFold2-predicted ion channel structures refer primarily to the most stable structures inferred from available templates—usually corresponding to the closed state of ion channels—rather than explicitly representing the thermodynamic global minimum. AlphaFold2 does not employ an explicit energy function or Markov chain Monte Carlo (MCMC) sampling, thereby lacking the capacity to explore multiple local minima across the free energy landscape. Additionally, the majority of protein structures in the PDB, which serve as templates for AlphaFold2, are determined via X-ray crystallography. This method preferentially captures thermodynamically stable conformations (global minimum or dominant states, such as the closed state of ion channels). Although the use of channel modulators in combination with cryo-EM or X-ray crystallography can potentially resolve alternative conformational states, the non-closed states of ion channel structures contribute only a limited portion of the database. Crucially, AlphaFold2’s template features and homologous sequence attention mechanisms further reinforce predictions toward dominant conformations, leading to systematic neglect of low-population conformations (e.g., open states of ion channels). Consequently, AlphaFold2’s static prediction paradigm fundamentally limits its ability to resolve dynamic structural transitions of ion channel proteins. Finally, AlphaFold2 struggles to accurately predict protein complexes, even though most ion channels operate as multimeric assemblies. And the inability to model interactions between ion channel proteins and ions, ligands, or other biomacromolecules hinders investigations into how these molecules modulate channel function. To address these shortcomings, researchers have often turned to molecular docking or molecular dynamics simulations to explore interactions between AlphaFold2-predicted protein structures and various ions, ligands, or other biomacromolecules. Nonetheless, these approaches have proven inadequate for multiple reasons. Docking and simulation studies indicate that AlphaFold2 frequently fails to recognize and position these molecules within the ion channel protein, cannot reliably capture low-energy conformations, and exhibits lower accuracy when predicting complex protein assemblies. Although AlphaFold-Multimer improved quaternary structure predictions, its performance remains inferior to that of single-chain predictions [16].

In response to these challenges, DeepMind developed AlphaFold3 (AF3), which substantially expands on the capabilities when compared with AlphaFold2 [17]. AlphFold3 incorporates several key innovations: Firstly, AlphaFold3 features an updated architecture employing a diffusion-based generative model to predict atomic coordinates directly, enhancing accuracy through improved conformational sampling. Unlike AlphaFold2’s gradient-based iterative refinement approach using FAPE loss, AlphaFold3 utilizes a diffusion model comprising two stages: forward diffusion (training) and reverse diffusion (sampling). In the forward diffusion process, noise is progressively added to known atomic coordinates across multiple timesteps, enabling the model to learn a denoising function that directly optimizes noise residuals via a simplified mean squared error (MSE) loss without explicit frame alignment. During reverse diffusion, AlphaFold3 begins with randomly initialized atomic coordinates, iteratively applying the learned denoising function to systematically reduce noise, refining structures from global arrangements down to fine atomic details. This multiscale denoising facilitates robust sampling across diverse conformational landscapes, effectively resolving AlphaFold2’s issue of becoming trapped in local minima. Additionally, the generative nature of the diffusion model allows AlphaFold3 to predict reasonable structures even with incomplete input information, significantly expanding its utility. Secondly, AlphaFold3 substantially broadens its predictive scope beyond single-protein targets to predict joint structures of complexes involving proteins, nucleic acids, small molecules, ions, and modified residues, exhibiting markedly improved performance in protein-ligand, protein-nucleic acid, and antibody-antigen complexes. For example, when modeling protein-ligand interactions, AlphaFold3 outperforms classical docking tools such as AutoDock Vina; in benchmark datasets like PoseBusters, it achieves higher accuracy and yields a larger fraction of protein-ligand pairs with root mean square deviations (RMSDs) below 2 Å [[17], [18], [19]]. When modeling protein-nucleic acid complexes, AlphaFold3 shows enhanced precision for RNA-protein and DNA-protein assemblies, often surpassing methods like RoseTTAFold and handling thousands of residues [[20], [21], [22]]. In addition, AlphaFold3 accurately represents covalent modifications, such as glycosylation and other post-translational changes, thereby facilitating detailed studies on protein regulation and function [17]. Finally, AlphaFold3 introduces a new confidence module that predicts atom-level and pairwise errors, offering valuable insights into final structure reliability. This module uses several advanced metrics, including the predicted Local Distance Difference Test (pLDDT), a per-residue confidence measure on a 0–100 scale where higher values signal closer agreement with true structures. These scores are often visualized through color coding for intuitive interpretation. Another metric, the Predicted Aligned Error (PAE), estimates positional errors between residues, with lower values indicating higher confidence. Finally, AlphaFold3 outputs two global confidence scores: the predicted Template Modeling score (pTM), which evaluates the overall accuracy of the global fold by comparing predicted and native structures, and the interface predicted Template Modeling score (ipTM), which assesses the reliability of interfacial arrangements in multimeric or complex structures. Higher scores imply more reliable predictions, with a pTM above 0.5 suggesting that the predicted fold closely resembles the native structure, and an ipTM above 0.8 pointing to high-quality and reliable subunit positioning within a complex [16,17]. Together, pTM and ipTM are particularly important for modeling ion channel complexes, as they help evaluate the correctness of assembly and subunit interface orientation.

By leveraging these capabilities, AlphaFold3 enables researchers to explore the physiological functions of ion channel complexes from a structural perspective. High-precision structural predictions can partly replace traditional experimental analyses and can be directly applied to structure-function investigations. Moreover, these innovations facilitate more accurate identification of critical binding interfaces in complexes and offer deeper insights into interactions between ion channels, biomacromolecules, and small-molecule ligands.

## 3 Basic analyses of structure-function relationships using AlphaFold3

### 3.1 General protocol

**Fig. 1:**
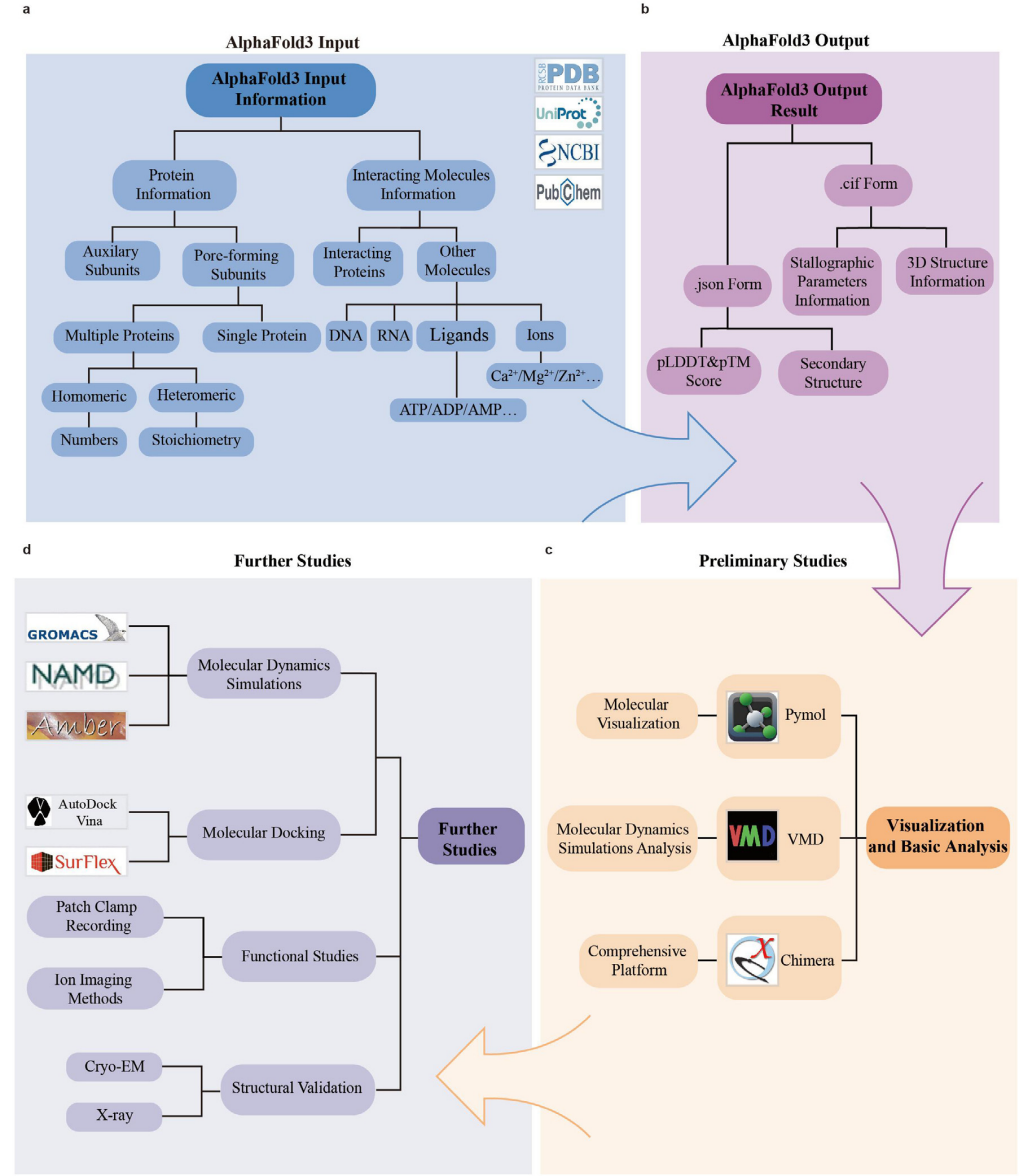
**A brief guideline for studies of structure-function relationships of ion channels using AlphaFold3.** (a) Guideline for input of ion channel complex to AlphaFold3. (b) The output of AlphaFold3 after building the structure of the ion channel complex. (c) Commonly used software for preliminary analysis of AlphaFold3 predicted structure of ion channel complex. (d) Commonly used methods for In-depth studies of ion channel complexes. (For interpretation of the references to colour in this figure legend, the reader is referred to the web version of this article.)

Here we present a general protocol for using AlphaFold3 to investigate the structure-function relationships of ion channel complexes (Fig. 1). Begin by compiling detailed information about the ion channel and any associated molecules (Fig. 1a). Relevant data, including amino acid sequences and small-molecule structures, can be accessed through databases such as UniProt (comprehensive protein sequences and functional details), the Protein Data Bank (PDB), National Center of Biotechnology Information (NCBI), PubChem, or custom repositories. Ion channels can exist as a single protein subunit with auxiliary proteins (e.g., mammalian voltage-gated calcium or sodium channels, which often associate with auxiliary subunits for trafficking or regulation [4]) or as multi-subunit complexes. In multi-subunit systems, channels may form homomeric assemblies like the tetrameric TRPV1, or heteromeric assemblies like the PKD1-PKD2 complex. Determining whether the assembly is homomeric or heteromeric, as well as establishing the correct stoichiometry, is crucial for accurate structure prediction.

Once the ion channel complex is defined, submitting all the information to the AlphaFold3 online server yields output files in JSON and CIF formats (Fig. 1b). JSON files provide details on confidence scores (pLDDT, pTM) and predicted secondary structures, while CIF files include crystallographic parameters and three-dimensional coordinates. Subsequent visualization and structure-function analyses can be performed using software such as PyMol, VMD, or Chimera (Fig. 1c). For more in-depth studies (Fig. 1d), molecular dynamics simulations can be employed using packages such as GROMACS, NAMD, Amber, CHARMM, or LAMMPS to explore the dynamic behavior and functional mechanisms of ion channels. In parallel, tools like AutoDock Vina or SurFlex can be used for docking analyses, particularly valuable in drug discovery or investigations into small-molecule modulation of ion channels. Finally, experimental methods, including patch-clamp electrophysiology, ion imaging and structural determination by cryo-EM and X-ray, can validate structure-based hypotheses by confirming channel function and ligand effects in vitro or in vivo.

### 3.2 Preliminary studies of ion channels using AlphaFold3

AlphaFold3 can be broadly applied in three areas: predicting structures of ion channel complexes, investigating molecular interactions, and evaluating channel variants.

#### 3.2.1 Predicting structures of ion channel complex

Before AlphaFold3, computational deep learning methods, such as AlphaFold2, RoseTTAFold2, and ESMFold, were extensively used to predict pore-forming subunits of ion channels [23]. However, most studies centered on single-protein pore-forming units (e.g., mammalian voltage-gated sodium channels Na_V_ or voltage-gated calcium channels Ca_V_) or symmetrical assemblies such as voltage-gated potassium channels (K_V_) and TRP channels [23,24]. In reality, many ion channels adopt more complex configurations: some form heteromeric assemblies (e.g., P2X heterotrimers and TRPC heterotetramers [25,26]), while others occur as heteromeric complexes like PKD1-PKD2 channel complex [27]. AlphaFold2 and related approaches often fail to capture entire multi-subunit structures in sufficient detail [28], whereas AlphaFold3 substantially improves predictions of these complex architectures, especially in heteromeric systems. Even homomeric ion channels can exhibit variable subunit compositions; for instance, TRPV3, typically regarded as a tetramer, can also form pentameric channels [29]. By enabling adjustments to the number of subunits and exploring alternative inputs, AlphaFold3 provides a more comprehensive view of ion channel quaternary structure, thereby advancing our understanding of their functional diversity.

#### 3.2.2 Investigating intermolecular interactions

Ion channels frequently function alongside auxiliary subunits and regulatory partners that modulate their activity. For example, voltage-gated sodium (Na_V_) and calcium (Ca_V_) channels often assemble with β subunits to facilitate channel trafficking [30], and mammalian sperm cation channels associate with multiple auxiliary proteins [31]. AMPA-type ionotropic glutamate receptors (AMPARs) also rely on auxiliary subunits to influence channel properties [32]. Beyond these subunits, many proteins directly interact with ion channels to modulate their functions. Calmodulin (CaM), the primary calcium sensor in eukaryotes, regulates a variety of channels (Ca_V_, Na_V_, K_V_, and TRP) [33], while calcium/calmodulin-dependent kinase II (CaMKII) is known to interface with channels such as Ca_V_, Na_V_, and NMDA-type glutamate receptors (NMDARs), positioning it as a key organizer of synaptic plasticity, learning, and memory [[34], [35], [36]]. Additionally, the neuronal calcium sensor Visinin-like protein-1 (VILIP1) can interact with P2X2 receptors and nicotinic acetylcholine receptors (nAChRs) [37,38]. AlphaFold3 offers a valuable platform for elucidating these protein-protein interactions at atomic resolution.

In addition to protein complexes, ion channels are influenced by various non-protein molecules. For instance, Ca^2+^ ions can modulate TRP channels, BK channels, calcium-activated chloride channels (TMEM16A), and P2X receptors [[39], [40], [41], [42]]. MicroRNAs have been implicated in activating TRPA1 and the 5-HTR2B/TRPV4 complex [43,44], while lipid molecules modulate mechanosensitive OSCA/TMEM63 channels, mechanosensitive YnaI channels, TRPC channels, and others [[45], [46], [47], [48]]. Predicting these interactions with AlphaFold3 allows for deeper insights into the network of molecules that regulate ion channel function.

#### 3.2.3 Evaluating channel variants

Ion channel dysfunction, commonly referred to as channelopathies, can lead to a diverse array of disorders in the nervous, cardiovascular, respiratory, endocrine, urinary, and immune systems. A substantial proportion of these pathologies stems from mutations in ion channel genes [1]. For instance, single-point mutations in Ca_V_1.2 result in Timothy Syndrome, characterized by life-threatening arrhythmias, structural heart defects, syndactyly, distinctive facial features, and autism spectrum disorders [49]. Widespread mutations have also been identified in Na_V_ and K_V_ channels, potentially causing neurodevelopmental and cardiovascular conditions [[50], [51], [52]], while TRPC channel mutations can lead to focal segmental glomerulosclerosis (FSGS) [53]. Furthermore, alternative splicing in ion channels plays an important role in modulating channel function and disease progression [54]; for example, N- and T-type voltage-gated calcium channel splice variants may differentially contribute to chronic pain [55], and certain Ca_V_1.3 variants show altered sensitivity to dihydropyridine inhibitors [56]. Likewise, TRP channels exhibit numerous splicing events that can affect their functional properties [57]. Analyzing these variants, which include both mutations and splice forms, within the context of the complete ion channel complex using AlphaFold3 can greatly improve our understanding of structure-function relationships and the molecular mechanisms behind channelopathies.

### 3.3 Key parameters for basic analyses

Visualization and basic analyses of ion channel structures can provide both qualitative and quantitative information for structure-function relationships. The list below includes many commonly assessed parameters.

**Geometric and structural metrics:** including Inter-Atomic Distances, Root Mean Square Deviation (RMSD), which measures overall structural stability or deviation from a reference conformation, Root Mean Square Fluctuation (RMSF), which quantifies the flexibility/mobility of individual residues over time, Angles and Dihedral Angles, and the Radius of the Channel Pore.

**Chemical bonding and interactions:** including hydrogen bonds (H-bonds), salt bridges/ionic interactions, π-π and cation-π interactions, and disulfide bonds.

**Surface and electrostatics:** including the surface charge distribution or electrostatic potential maps, which contribute to ion selectivity, ion permeation, gating, and potential binding sites for ligands or modulators; solvent accessible surface area (SASA), which reflects how much of the protein is exposed to solvent and can be relevant to protein stability or ligand accessibility.

**Ion conduction pathways:** including the pore radius profiles, which characterize the shape and possible gating bottlenecks of the channel; and ion occupancy and positions, which show where ions reside during conduction.

**Dynamics and conformational transitions:** including the conformational ensembles, which identify different states like open versus closed; and cross-correlation analyses, which help to reveal the collective movements of domains or subunits.

**Allosteric and cooperative effects:** including the subunit interfaces in multimeric channels and interactions at subunit boundaries, which can modulate gating or ion permeation; and the allosteric sites, which are regions away from the main active/pore site that, when bound by a modulator, induce functional changes.

**Residue/atomic-level insights:** including the key/hotspot residues/atoms, conserved motifs, mutations, and variants that are crucial for ion channel functions.

### 3.4 Tools for basic analyses

PyMOL, VMD, and Chimera are commonly used for fundamental analyses of three-dimensional channel structures, enabling detailed visualization, manipulation, and assessment of structural features. Their capabilities support hypothesis generation and guide experimental design.

PyMOL is one of the most frequently used molecular visualization programs and accommodates a wide range of file formats for proteins, nucleic acids, and other biomolecules. Its strengths lie in generating high-quality, publication-ready images and providing a Python-based scripting environment. Researchers use PyMOL to visualize molecular architectures, highlight specific residues or domains, create molecular animations, and analyze protein-ligand interactions. Although the graphical interface is relatively intuitive, advanced users often employ scripting to automate workflows and perform more intricate analyses.

Chimera and ChimeraX are adaptable platforms for interactive visualization and analysis of molecular structures and associated data, including density maps, sequence alignments, and docking results. Tasks supported by Chimera/ChimeraX include comparative protein modeling, electron microscopy map fitting, and structural annotation. Its user-friendly graphical interface appeals to a broad range of researchers, while built-in Python scripting and third-party extensions provide customizable options for complex structural biology analyses. In addition to producing elegant molecular figures, Chimera/ChimeraX often serves as a comprehensive platform for integrative modeling and hypothesis generation.

VMD was originally released in 1995 by the University of Illinois at Urbana-Champaign. It offers powerful and flexible. It specializes in the visualization and analysis of molecular dynamics simulation data, including the molecular dynamics trajectories, RMSD calculations, electrostatic potential mapping, and hydrogen bond identification. VMD excels at handling large datasets, allowing researchers to track the time-dependent behavior of proteins, membranes, and other macromolecular complexes. Its integration with molecular dynamics packages like NAMD and GROMACS facilitates a seamless workflow for exploring dynamic molecular processes.

## 4 Further studies

The predicted structures of ion channels can be extended to more detailed investigations aimed at clarifying their functional mechanisms. Analyses of conformational dynamics and transitions can help in identifying different functional states, tracking the pathways between these states, quantifying potential energy barriers, and revealing collective movements of residues, domains, or subunits. Thermodynamic and kinetic assessments, such as potential energies of ions or ligands, free energy landscapes (FEL), and potential of mean force (PMF), can quantitatively explain the operational principles of these channels. Experimental approaches, including electrophysiological measurements and imaging techniques, can then verify the hypotheses generated by computational studies. Generally, these in-depth analyses can be grouped into three categories: molecular dynamics simulations, molecular docking studies, and functional investigations.

### 4.1 Molecular dynamics simulations

Molecular dynamics (MD) simulation is a cornerstone technique for examining the dynamic behavior of biomolecules, including ion channels, by numerically solving Newton’s equations of motion. Key simulation components include the choice of force field, system setup, integration algorithms, boundary conditions, and temperature/pressure controls. Widely used software packages in biomolecular system each offer distinct advantages: GROMACS is known for its speed and scalability, supporting multiple force fields and parallel computing architectures; NAMD excels at simulating large membrane proteins; and other programs such as AMBER, CHARMM, OpenMM, and Desmond are also routinely employed for biomolecular systems.

Applying MD simulations to ion channels can yield insights into structural dynamics, conformational transitions underlying gating mechanisms, ion permeation and selectivity, drug or ligand binding sites and kinetics, and the impact of protein or lipid components on channel function. For instance, Sun et al. used AlphaFold2 to predict the human Ca_V_ structure, refined it with cryo-EM data, and investigated gating mechanisms using GROMACS [58]. Ngo et al. guided AlphaFold2 to predict hERG channel conformations and explored gating behavior via MD in Amber22 [59], while Giese et al. combined AlphaFold2 predictions with MD simulations to analyze TMC1/2 complexes bound to CIB2/3 [60]. Given AlphaFold3’s enhanced ability to predict complex multimeric systems, we anticipate even greater potential for advancing ion channel research through similar MD-based approaches.

### 4.2 Molecular docking

Molecular docking is a pivotal computational technique for predicting and analyzing how small molecules, ligands, and other biomacromolecules interact with their target proteins, including ion channels. It is widely used in drug discovery to explore ligand specificity and elucidate the molecular basis of ion channel modulation by small molecules. Common software tools include AutoDock Vina, DOCK and SurFlex. Compared with MD simulations, docking focuses on static protein-ligand interactions and is often considered a simplified, high-efficiency approach to screen potential drug candidates.

For example, Wang et al. used cryo-EM data, docking models, and experimental validation to identify two distinct binding sites of small-molecule drugs on the polycystin-2 channel [61]. Similarly, Sheokand et al. [62] conducted molecular docking to investigate marine compounds targeting voltage-gated calcium channel subunits (α_2_δ-1) for anti-epileptic drug discovery, and Wei et al. [63] applied a machine learning-assisted docking strategy to screen for novel TRPV1 modulators. With its ability to predict the structure of complex ion channel assemblies, AlphaFold3 can facilitate more refined docking analyses by providing accurate models of ion channel-ligand interactions, thus further advancing both fundamental research and therapeutic discovery.

### 4.3 Functional studies

After performing computational analyses on ion channel complexes, functional studies—including electrophysiology, imaging techniques, and high-resolution structure determination—are necessary to confirm the validity of theoretical predictions. Patch-clamp methods remain the gold standard for ion channel characterization, with both single-channel and whole-cell configurations widely used in academic settings to investigate gating mechanisms, often in combination with molecular biology. Automated patch-clamp systems provide a high-throughput option for industrial drug discovery and ion channel screening. Imaging techniques, such as calcium and potassium ion imaging, also play a major role in examining channel function; advances in the fluorometric imaging plate reader (FLIPR) system (usually for calcium assays) and ion channel readers (often for potassium assays) facilitate high-throughput analysis. For example, Zhang et al. [64] employed FLIPR to screen T-type calcium channel modulators, and Montalbano et al. [65] used an ion channel reader for high-throughput clone screening of overexpressed hERG1 and K_V_1.3 potassium channels. Meanwhile, cryo-electron microscopy (cryo-EM), X-ray crystallography and nuclear magnetic resonance (NMR) have become the leading approaches for experimentally resolving ion channel structures. Such experimental work not only verifies but also refines computational models, while the predictions from AlphaFold and other tools can streamline and guide structure determination. Although computational methods have advanced significantly, especially for ion channel research, their outcomes still require thorough experimental validation, underlining the indispensable nature of functional and structural studies.

### 4.4 Summary of software and databases

Here we provide an overview of commonly used software and databases relevant to structure-function studies of ion channel complexes (Table 1). Although this table highlights key resources, it is not exhaustive; many additional tools are also widely utilized in the field, and the information here serves primarily as a reference.

**Table 1:** **Commonly used software and databases for the structure-function studies of ion channel complex**.

Name	Introduction	Key resources
AlphaFold	Early neural network-based approach for protein structure prediction, demonstrated at CASP13. It laid the groundwork for subsequent improvements in accuracy and speed.	Ref. [12]
AlphaFold2	A major leap in protein structure prediction, providing near-experimental accuracy for single-chain proteins. Published after CASP14, it uses deep learning and evolutionary data to predict structures without relying on known templates.	Ref. [14] https://alphafold.com/
AlphaFold-Multimer	An extension of AlphaFold2 for predicting protein complexes.	Ref. [16] https://github.com/google-deepmind/alphafold/blob/main/docs/technical_note_v2.3.0.md
AlphaFold3	A newer iteration that expands AlphaFold’s scope to non-protein molecules and complex multimolecular interactions, incorporating refined algorithms and confidence metrics.	Ref. [17] https://alphafoldserver.com
RoseTTAFold2	A successor to RoseTTAFold with improvements in accuracy comparable to AlphaFold2, developed by the Baker Lab. Often used in academic settings.	Ref. [21] https://github.com/uw-ipd/RoseTTAFold2
RoseTTAFold All-Atom	RoseTTAFold All-Atom is an advanced version of the RoseTTAFold protein structure prediction system that refines atomic details, enhancing accuracy for tasks such as side-chain modeling, multimeric interfaces, and ligand binding predictions.	Ref. [20]
ESMFold	A fast protein structure predictor developed by Meta AI, leveraging large language models. Offers significantly reduced inference time while maintaining accuracy similar to AlphaFold2 for many single-protein cases.	Ref. [66] https://github.com/facebookresearch/esm
UniProt	A comprehensive, curated database of protein sequence and functional information, widely used as a primary reference for protein annotations and identifiers.	https://www.uniprot.org/
RCSB PDB	A major repository for experimentally determined 3D biomolecular structures, including proteins, nucleic acids, and complex assemblies.	https://www.rcsb.org/
NCBI	The U.S. National Center for Biotechnology Information, offering a range of databases (e.g., GenBank, PubMed) and bioinformatics tools for molecular biology and genomics research.	https://www.ncbi.nlm.nih.gov/
PubChem	A free online database of chemical molecules, including bioactive compounds, drugs, and small molecules, widely used in cheminformatics and drug discovery.	https://pubchem.ncbi.nlm.nih.gov/
PyMOL	A popular molecular visualization tool built on Python, known for producing publication-quality images and supporting scripting for batch analyses. Widely employed for highlighting key residues, domains, or interactions.	https://pymol.org/
VMD	A comprehensive program for visualizing and analyzing MD trajectories, originally released by the University of Illinois at Urbana-Champaign. Integrates seamlessly with NAMD and can handle large simulation datasets.	https://www.ks.uiuc.edu/Research/vmd/
Chimera	A highly adaptable molecular visualization suite supporting density maps, sequence alignments, and docking results. Offers an intuitive interface and Python-based scripting, making it valuable for integrative modeling and advanced figure preparation.	https://www.cgl.ucsf.edu/chimera/
ChimeraX	A next-generation molecular visualization program from the Resource for Biocomputing, Visualization, and Informatics (RBVI), following Chimera.	https://www.cgl.ucsf.edu/chimerax/
Amber	A suite of MD programs featuring the AMBER force fields, popular for simulating proteins and nucleic acids. Known for ease of use, but can be slower compared with newer optimized codes.	https://ambermd.org/tutorials/
CHARMM	A well-established MD engine paired with the CHARMM force fields, offering extensive capabilities for protein, lipid, and nucleic acid simulations. Commonly employed in academic research for advanced modeling.	https://academiccharmm.org/
OpenMM	An open-source MD toolkit from the Pande Lab, designed for GPU acceleration. Often used as a library within Python, making it highly flexible for custom simulations and integrative modeling.	https://openmm.org/
AutoDock	A free, widely cited molecular docking software suite used to predict how ligands (often small molecules) bind to macromolecular targets. Good for introductory docking studies.	https://autodock.scripps.edu/
AutoDock Vina	An enhanced version of AutoDock offering faster runtime and improved accuracy in docking predictions, frequently used for virtual screening and ligand optimization.	https://vina.scripps.edu/
Surflex	A docking tool designed for protein-ligand interactions, often employed in drug discovery workflows. Uses a proprietary scoring function to predict binding poses.	Ref. [67]

## 5 Case studies

Here, the P2X family of receptors and voltage-gated calcium channel Ca_V_2.1 serve as examples of how AlphaFold3 can advance our understanding of ion channel structure-function relationships.

### 5.1 Prediction of the human P2X4 receptor using AlphaFold3

P2X receptors are ATP-gated cation channels expressed in various tissues, where they contribute to synaptic transmission, muscle contraction, platelet aggregation, inflammation, macrophage function, cell proliferation, and both neuropathic and inflammatory pain [68]. Elucidating P2X channel structures enhances insights into their gating mechanisms and aids in identifying potential therapeutic targets for inflammation, pain, and cardiovascular diseases.

**Fig. 2:**
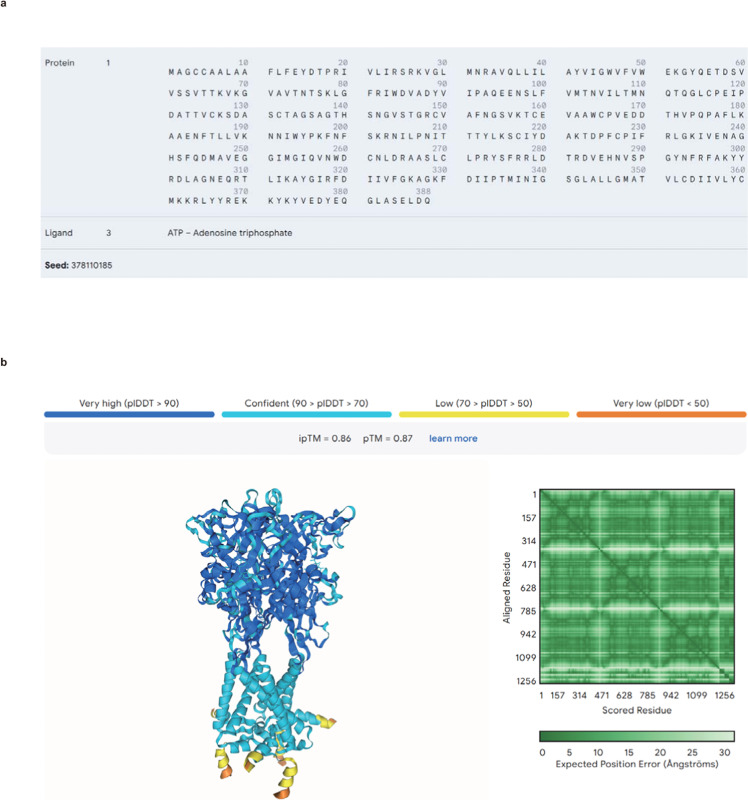
**AlphaFold3 input and output.** (a) Input: Amino acid sequences of the human P2X4 receptor (Uniprot ID: Q99571) and three ATP molecules are submitted to AlphaFold3. (b) Output: The predicted structure is visualized using a color-coded confidence map based on predicted Local Distance Difference Test (pLDDT) scores. Residues with pLDDT > 90 are shown in blue (very high confidence), 70 < pLDDT < 90 in cyan (high confidence), 50 < pLDDT < 70 in yellow (low confidence), and pLDDT < 50 in orange (very low confidence). Additionally, the Predicted Aligned Error (PAE) matrix is displayed, showing the expected position error between pairs of residues. The matrix is color-coded from green (0 Å error, highest confidence) to white (30 Å error, lowest confidence). The structure's global confidence metrics are ipTM = 0.86 and pTM = 0.87. Figure adapted from AlphaFold Server and Ref. [17]. Reproduced under the terms of the CC-BY license. Copyright 2024, published by Springer Nature. Reproduced with permission. (For interpretation of the references to colour in this figure legend, the reader is referred to the web version of this article.)

The P2X4 receptor can function as a homotrimeric channel that senses extracellular ATP concentrations. The crystal structures of the P2X4 receptor in closed, ATP-bound, and apo states have been resolved [69], revealing significant conformational changes tied to ATP binding, which acts as a key process for channel gating. However, AlphaFold2 has limited capability in modeling such ligand-bound complexes. In contrast, AlphaFold3 enables reliable prediction of the P2X4-ATP complex using its online server (Fig. 2a). The resulting model displays high pTM value (0.87) and ipTM value (0.86), reflecting strong confidence and consistency with available experimental data (Fig. 2b).

### 5.2 Comparison of P2X4 receptor monomer structures predicted by AlphaFold2 and AlphaFold3

**Fig. 3:**
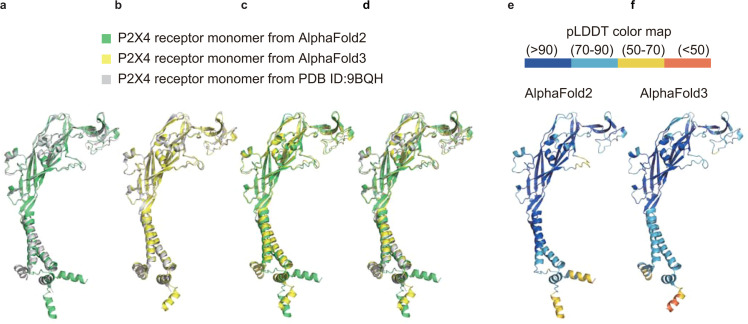
**Comparison of P2X4 receptor structures predicted by AlphaFold2 and AlphaFold3.** (a-d) Structural comparisons of the P2X4 receptor monomer (UniProt ID: Q99571) predicted by AlphaFold2 (green), AlphaFold3 (yellow), and experimentally resolved structure (gray; PDB: 9BQH). Structural alignments are shown for: (a) AlphaFold2 prediction versus experimental structure; (b) AlphaFold3 prediction versus experimental structure; (c) AlphaFold2 prediction versus AlphaFold3 prediction; (d) Overlay of AlphaFold2 prediction, AlphaFold3 prediction, and experimental structure. (e, f) pLDDT color maps for structures predicted by AlphaFold2 (e) and AlphaFold3 (f). Residues with pLDDT > 90 are shown in blue (very high confidence), 70 < pLDDT < 90 in cyan (high confidence), 50 < pLDDT < 70 in yellow (low confidence), and pLDDT < 50 in orange (very low confidence). Figure adapted from AlphaFold Server and Ref. [17]. Reproduced under the terms of the CC-BY license. Copyright 2024, published by Springer Nature. Reproduced with permission. (For interpretation of the references to colour in this figure legend, the reader is referred to the web version of this article.)

To evaluate the performance of AlphaFold3 relative to AlphaFold2, we predicted the structure of homoP2X4 monomer using both algorithms (Fig. 3a-d; Table 2) and compared the results with the available experimental structures using RMSD values as a metric of structural similarity (Table 3). The AlphaFold3-predicted model shows greater similarity to the experimental structure of the apo state/close state compared to that predicted by AlphaFold2. In terms of prediction confidence, both AlphaFold2 and AlphaFold3 yielded comparable per-residue confidence scores based on the pLDDT color map and average pLDDT values (Fig. 3e-f; Table 2). However, AlphaFold3 achieved a higher predicted Template Modeling (pTM) score (Table 2), suggesting a more reliable overall fold.

**Table 2:** **Statistical comparison of P2X4 receptor structures predicted by AlphaFold2 and AlphaFold3**.

		AlphaFold2	AlphaFold3
Average pLDDT	No. 1	86.76	84.51
	No. 2	87.19	84.57
	No. 3	88.04	84.48
	No. 4	87.26	84.42
	No. 5	87.28	84.22
pTM	No. 1	0.72	0.81
	No. 2	0.75	0.8
	No. 3	0.76	0.86
	No. 4	0.76	0.8
	No. 5	0.76	0.8

**Table 3:** **Comparison of P2X4 receptor structures predicted by AlphaFold2 and AlphaFold3 with experimental structures**.

	AlphaFold2	AlphaFold3
RMSD to PDB ID: 9C48 (ATP-bound desensitized state)	1.242	1.281
RMSD to PDB ID: 8BQH (apo state; closed state)	1.109	0.614
RMSD to PDB ID: 8BQI (BAY-1797-bound state)	1.172	0.869

In summary, while both AlphaFold2 and AlphaFold3 provide high-quality predictions for single-protein structures, AlphaFold3 exhibits a slight advantage in accurately modeling P2X receptor monomers.

### 5.2 Comparison of P2X4 receptor monomer structures predicted by AlphaFold2 and AlphaFold3

**Fig. 4:**
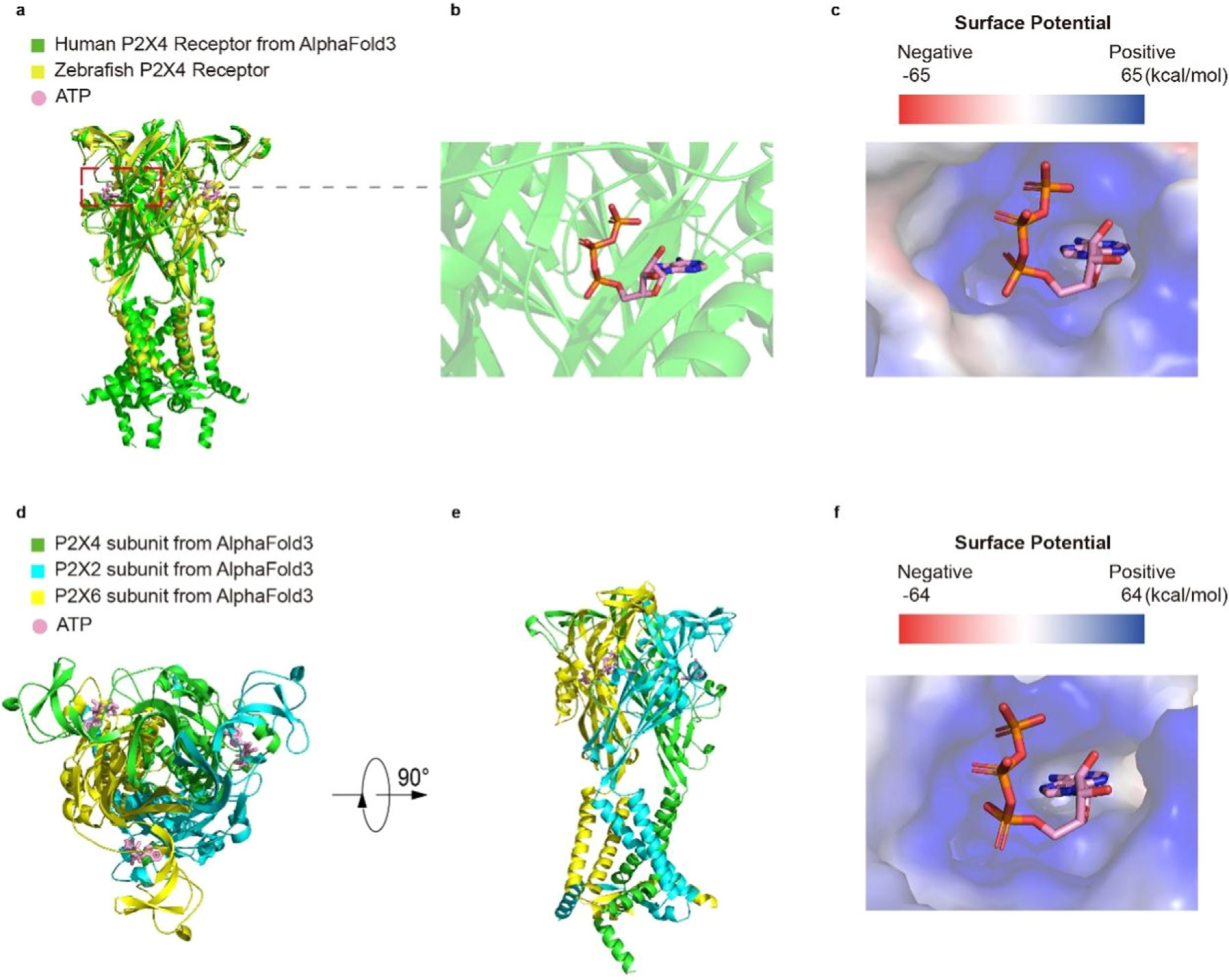
**Preliminary analyses of P2X receptors.** (a) Structure comparison of AlphaFold3 predicted human P2X4 receptor (green; Uniprot ID: Q99571, ipTM = 0.86, pTM = 0.87) and zebrafish P2X4 receptor structure (yellow; PDB ID: 4DW1). All structures are shown as cartoons in side view. (b) Close-up view of ATP binding site in AlphaFold3 predicted human P2X4 receptor. The ATP molecule is color-coded by atom type: the carbon backbone is shown in pink, oxygen atoms in red, and nitrogen atoms in blue. (c) Surface analyses of electrostatic potential for ATP-bound human P2X4 receptor, according to Coulomb’s law. Negative, neutral, and positive potentials are colored in red, white, and blue, respectively. The electrostatic potential surface is mapped onto the protein surface, ranging from –65 kcal/mol (red) to +65 kcal/mol (blue). (d, e) The top view (d) and side view (e) of the AlphaFold3 predicted P2X2-P2X4-P2X6 heterotrimer. P2X2 (UniProt ID: Q9UBL9) is colored as cyan, P2X4 (Uniprot ID:Q99571) is colored as green, and P2X6 (UniProt ID: O15547) is colored as yellow. ipTM = 0.72, pTM = 0.71. (f) Surface analyses of electrostatic potential for ATP-bound P2X2-P2X4-P2X6 heterotrimer, according to Coulomb’s law. The electrostatic potential surface is mapped onto the protein surface, ranging from –64 kcal/mol (red) to +64 kcal/mol (blue). Figure adapted from AlphaFold Server and Ref. [17], and further processed using PyMOL. Reproduced under the terms of the CC-BY license. Copyright 2024, published by Springer Nature. Reproduced with permission. (For interpretation of the references to colour in this figure legend, the reader is referred to the web version of this article.)

The modeled human P2X4 receptor displays a trimeric, cup-shaped architecture, with each subunit featuring two transmembrane helices and an extracellular domain containing extensive hydrophilic and glycosylated regions. Three ATP molecules bind to the ectodomains of the subunits, inducing conformational changes necessary for opening of P2X4 receptors. In the transmembrane region, six helices arrange in an iris-like motion upon activation. Superimposing the zebrafish and human P2X4 structures yields an RMSD of 0.961 Å, indicating minimal structural divergence (Fig. 4a, b). Detailed analyses of the structures using PyMOL can reveal several key amino acid residues that constitute the charged ATP binding pocket (Fig. 4c). These preliminary studies align with previous findings [70], indicating that the predicted human P2X4 structure is reliable. Moreover, P2X4 can form heterotrimers with P2X2, and P2X6 [25]. Modeling these heterotrimeric complexes via AlphaFold3 offers a route to studying their functional implications (Fig. 4d-f), which has been more challenging with earlier approaches like AlphaFold2.

### 5.3 Studies of P2X receptor interactions with regulatory proteins and ions

**Fig. 5:**
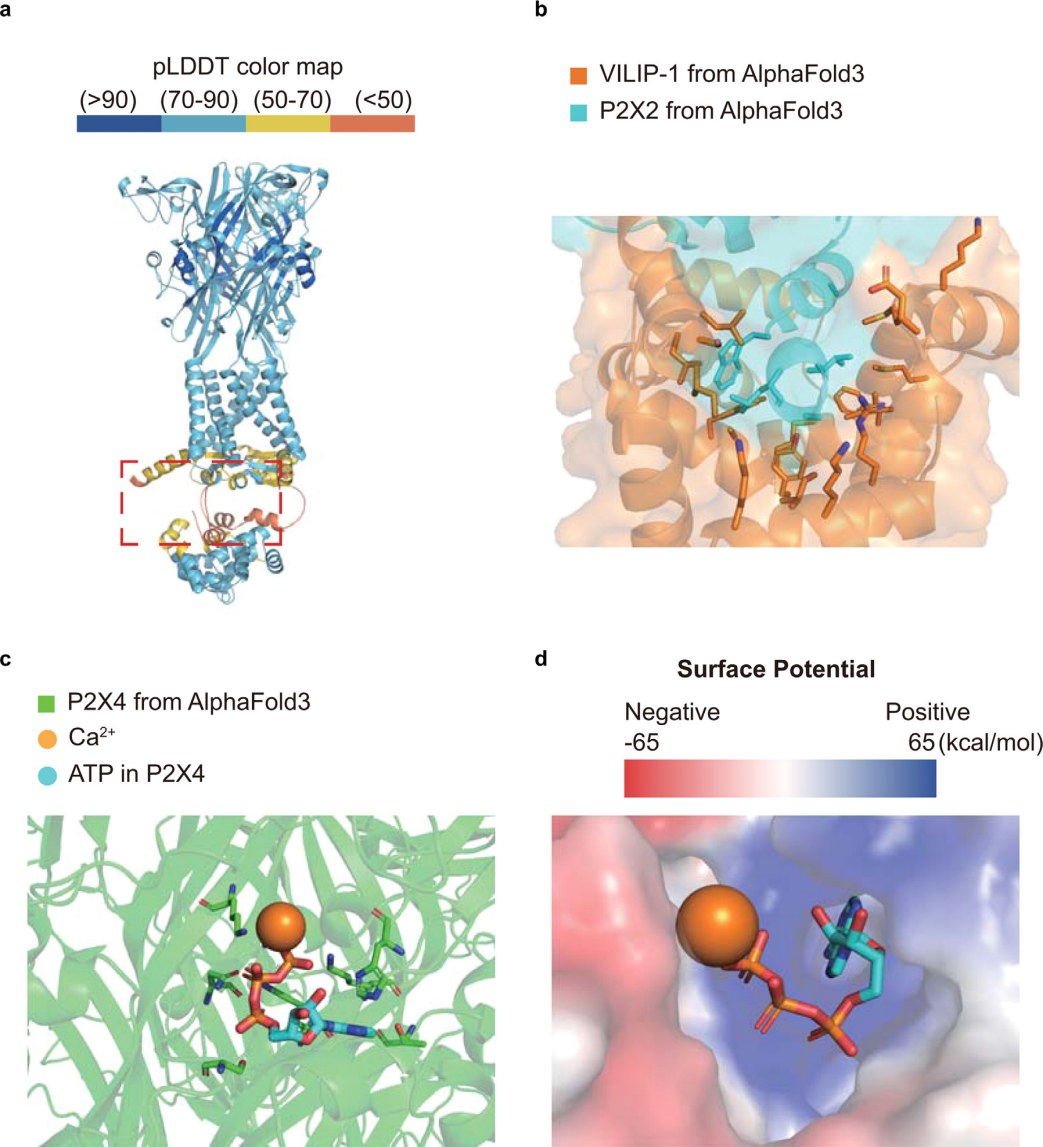
**Preliminary studies of P2X receptor interactions with regulatory proteins and ions.** (a) Predicted pLDDT map for the homoP2X2 (UniProt ID: Q9UBL9)/VILIP-1 (UniProt ID: P62760) complex, as generated by AlphaFold3. Residues with pLDDT > 90 are shown in blue (very high confidence), 70 < pLDDT < 90 in cyan (high confidence), 50 < pLDDT < 70 in yellow (low confidence), and pLDDT < 50 in orange (very low confidence). ipTM = 0.6, pTM = 0.64. (b) Close-up view of the interaction interface in the homoP2X2/VILIP-1 complex, with key residues shown in stick. P2X2 and VILIP-1 are colored cyan and orange, respectively. (c) Predicted homoP2X4-Ca^2+^-ATP complex, highlighting binding residues in stick. The P2X4 subunits are shown in green, calcium ions in orange, and ATP is depicted in atomic colors: the carbon backbone in cyan, oxygen atoms in red, and nitrogen atoms in blue. ipTM = 0.86, pTM = 0.87. (d) Surface electrostatic potential pocket of the Ca^2+^-ATP binding site in the homoP2X4 structure. The electrostatic potential surface is mapped onto the protein surface, ranging from –65 kcal/mol (red) to +65 kcal/mol (blue). Figure adapted from AlphaFold Server and Ref. [17], and further processed using PyMOL. Reproduced under the terms of the CC-BY license. Copyright 2024, published by Springer Nature. Reproduced with permission. (For interpretation of the references to colour in this figure legend, the reader is referred to the web version of this article.)

P2X receptors can also interact with various modulatory proteins and ions. For example, VILIP-1 is reported to interact with P2X2 receptors, forming a signaling complex that enhances ATP sensitivity and increases peak currents [37]. AlphaFold3 can model the homoP2X2/VILIP-1 complex (Fig. 5a, b), and subsequent molecular dynamics simulations may help refine the binding interface while revealing intracellular regulatory mechanisms that affect channel kinetics. However, the predicted binding interface between P2X2 and VILIP-1 shows low accuracy (Fig. 5a), which may affect the confidence in the interaction prediction. This limitation of AlphaFold3 highlights its inability to generate highly accurate predictions in the absence of structural templates. Additionally, P2X receptors are reported to be modulated by ATP bound to divalent cations [42]. AlphaFold3 can model the homoP2X4-Ca^2+^-ATP complex (Fig. 5c, d) to study the roles of cations in the gating mechanism of P2X receptors.

### 5.4 Studies of P2X receptor variants

**Fig. 6:**
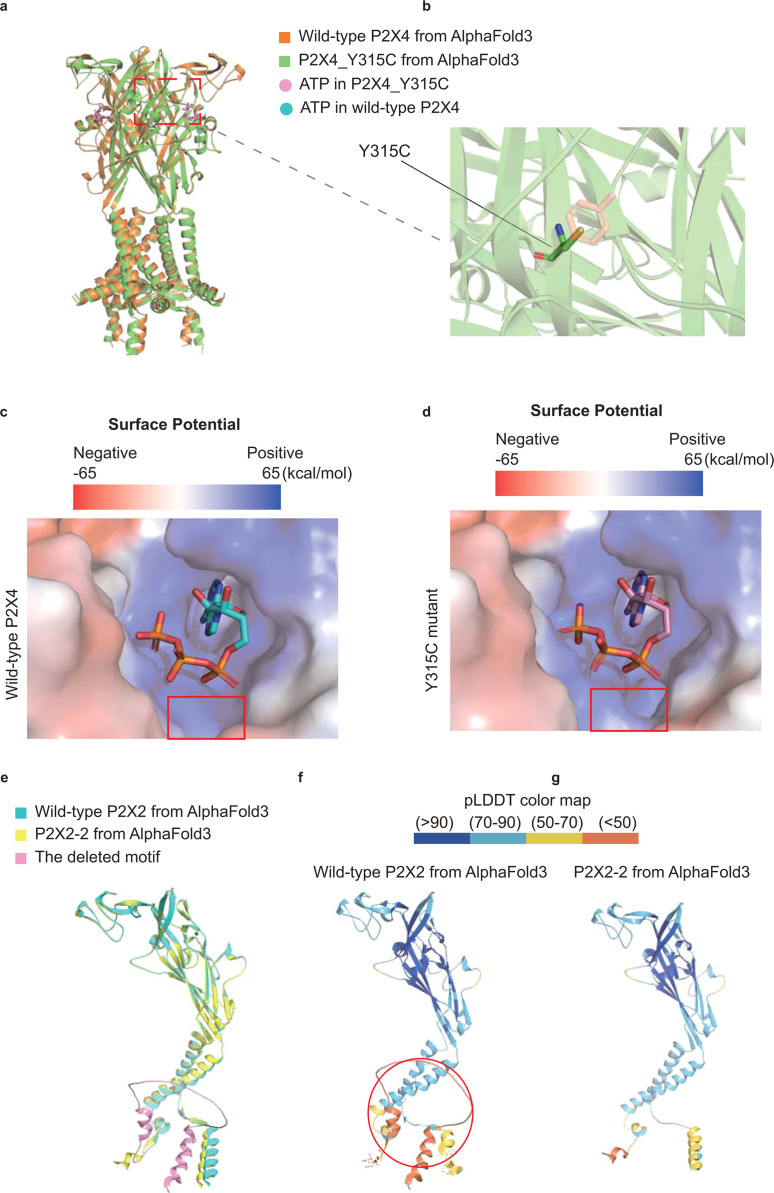
**Studies of P2X receptor variants.** (a) Side view of the wild-type P2X4 structure (orange, with ATP in blue) and the Y315C mutant (green, with ATP in pink; ipTM = 0.81, pTM = 0.83), with the ATP-binding region outlined by a rectangle. (b) Close-up view of the ATP binding site in the Y315C mutant. Residue 315 is shown in stick representation: the wild-type tyrosine (Y315) is rendered semi-transparent in orange, while the mutated cysteine (C315) is shown opaque in green. (c, d) Surface electrostatic potential at the ATP binding site for wild-type P2X4 (c) and P2X4_Y315C mutant (d). The electrostatic potential surface is mapped onto the protein surface, ranging from –65 kcal/mol (red) to +65 kcal/mol (blue). The primary difference compared to wild-type P2X4 is highlighted by a rectangle, showing a reduced positively charged region in the mutant structure. (e) AlphaFold3 predicted monomer of wild-type P2X2 (ipTM = 0.74, pTM = 0.74) and P2X2-2 splicing variant (ipTM = 0.77, pTM = 0.79). Deleted motif in P2X2-2 splicing variant is colored as pink. (f, g) pLDDT color map for wild-type P2X2 (f) and P2X2-2 splicing variant (g) as predicted by AlphaFold3. The deleted motif is circled. Residues with pLDDT > 90 are shown in blue (very high confidence), 70 < pLDDT < 90 in cyan (high confidence), 50 < pLDDT < 70 in yellow (low confidence), and pLDDT < 50 in orange (very low confidence). Figure adapted from AlphaFold Server and Ref. [17], and further processed using PyMOL. Reproduced under the terms of the CC-BY license. Copyright 2024, published by Springer Nature. Reproduced with permission. (For interpretation of the references to colour in this figure legend, the reader is referred to the web version of this article.)

Mutations and splicing of P2X receptors may disrupt the channel gating and contribute to pathological conditions. For instance, the loss-of-function Y315C mutation in P2X4 has been associated with increased compliance pulse pressure [71]. Additionally, a P2X2 receptor splice variant with a 207 bp deletion (P2X2-2) in the intracellular C-terminus has been reported to show significant differences in desensitization time constants and steady-state currents in the continuous presence of ATP when compared with the wild type [72]. By modeling the Y315C mutant and P2X2 splicing variant (P2X2-2) in AlphaFold3 and comparing it with the wild-type structure, researchers can use basic analytical tools (e.g., PyMOL) to evaluate the impact of these alterations (Fig. 6). Preliminary observations indicate an increased distance between residue Y315C and ATP (Fig. 6a-d), consistent with the previous study [71]. Interestingly, the deleted motif in the P2X2 splice variant exhibits a notably low pLDDT score (Fig. 6e-g) and corresponds to a domain that also remains unresolved in comparable experimental structures. Consequently, the overall confidence scores (ipTM and pTM) of the P2X2-2 splice variant prediction are higher than those of the wild-type P2X2. This is similar to the prediction of the interaction between P2X and VILIP-1, where the lack of a template results in lower prediction accuracy for specific domains. Additional analyses, such as molecular docking of ATP with the mutant receptor and functional studies, may provide further insight into how mutations and splicing variants contribute to the alteration of function.

### 5.5 Prediction of Ca_v_2.1 channel complex using AlphaFold3

Voltage-gated calcium channels (VGCCs) of the Ca_V_2.1 subtype are primarily localized at presynaptic terminals and somatodendritic membranes, where they play a critical role in mediating calcium influx and triggering neurotransmitter release. The Ca_V_2.1 channel complex typically comprises a pore-forming α_1A_ subunit, an intracellular β auxiliary subunit that contributes to membrane trafficking and channel regulation, and an extracellular α_2_δ auxiliary subunit [4].

**Fig. 7:**
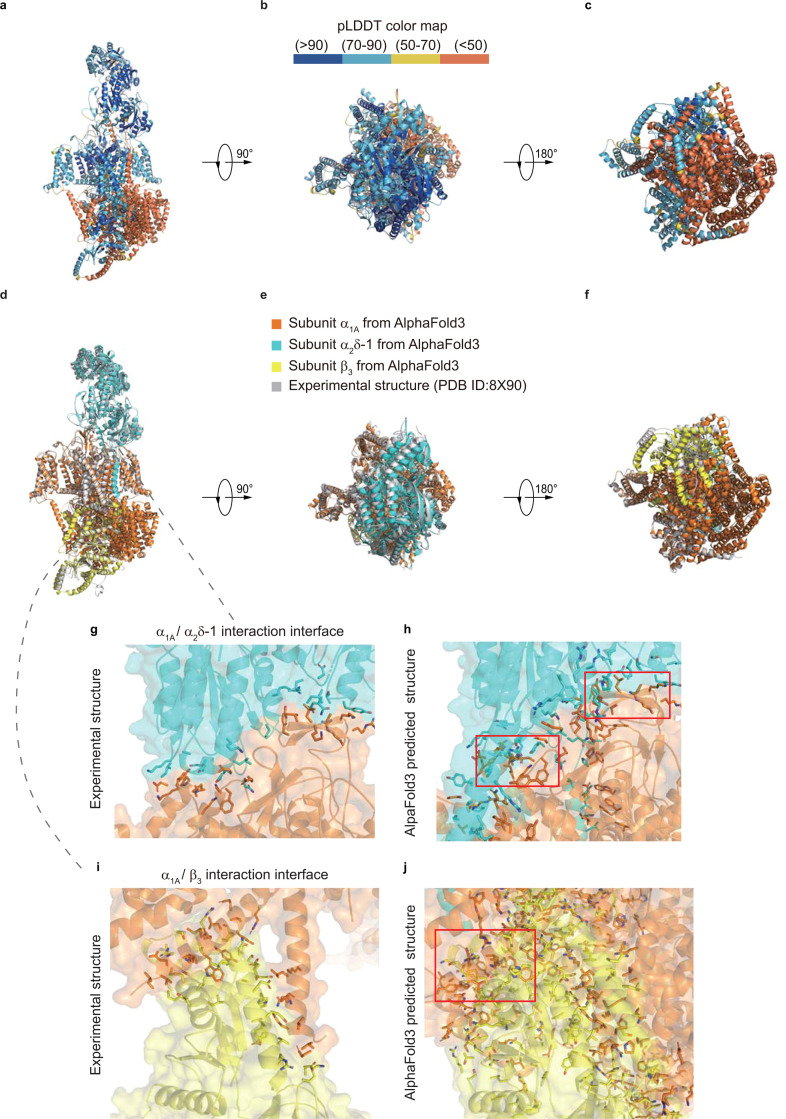
**AlphaFold3-predicted Ca_V_2.1 channel complex.** (a-c) pLDDT color map for the AlphaFold3-predicted Ca_V_2.1 channel complex (ipTM = 0.72, pTM = 0.68) in side view (a), top view (b) and bottom view (c). Residues with pLDDT > 90 are shown in blue (very high confidence), 70 < pLDDT < 90 in cyan (high confidence), 50 < pLDDT < 70 in yellow (low confidence), and pLDDT < 50 in orange (very low confidence). (d-f) Structural comparison between the AlphaFold3-predicted human Ca_V_2.1 channel complex structure and the experimentally resolved Ca_V_2.1 channel complex structure (gray; PDB ID: 8X90). The AlphaFold3-predicted structure is shown with the α_1A_ subunit (UniProt ID: O00555) in orange, the α_2_δ−1 subunit (UniProt ID: P54289) in cyan, and the β_3_ subunit (UniProt ID: P54284) in yellow. All structures are represented as cartoons in side view (d), top view (e), and bottom view (f). (g, h) Close-up views of the interaction interface between the α_1A_ and α_2_δ-1 subunits in the experimental structure (g) or in the AlphaFold3-predicted structure (h), highlighting key interacting residues shown as sticks, with the same area outlined by a rectangle. (i, j) Close-up views of the interaction interface between the α_1A_ and β_3_ subunits in the experimental structure (i) or in the AlphaFold3-predicted structure (j), highlighting key interacting residues shown as sticks, with the same area outlined by a rectangle. Figure adapted from AlphaFold Server and Ref. [17], and further processed using PyMOL. Reproduced under the terms of the CC-BY license. Copyright 2024, published by Springer Nature. Reproduced with permission. (For interpretation of the references to colour in this figure legend, the reader is referred to the web version of this article.)

Using AlphaFold3, we predicted the ternary complex structure of the human Ca_V_2.1 channel, consisting of α_1A_, β_3_, and α_2_δ-1 subunits (Fig. 7a-f), with pTM = 0.68 and ipTM = 0.72. The predicted structures of α_1A_ and α_2_δ−1 showed high confidence levels and structural consistency when compared with the available cryo-EM structure of the Ca_V_2.1 complex (Fig. 7a-f). However, a substantial portion of the β_3_ subunit remains unresolved in the cryo-EM data, and the corresponding region in the AlphaFold3 prediction exhibited very low confidence scores (Fig. 7a-f). This limitation highlights the current challenge AlphaFold3 faces when predicting structures of regions with insufficient training data or lacking structural templates.

Additionally, we examined the binding interfaces between α_1A_/α_2_δ-1 and α_1A_/β_3_. The predicted α_1A_/α_2_δ-1 interface in the AlphaFold3 model closely matches the experimentally validated interface in the cryo-EM structure (Fig. 7g, h), confirming the reliability of AlphaFold3 in reproducing known protein-protein interactions. Likewise, the previously characterized α1A/β3 binding interface is also recapitulated in the AlphaFold3 prediction (Fig. 7i, j). Notably, AlphaFold3 also resolves previously missing segments, revealing novel contact regions not observed in the experimental structure (Fig. 7g-j). While the accuracy of these newly predicted interfaces remains to be experimentally validated, they offer valuable hypotheses for future biochemical and structural investigations. These findings underscore both the promise and current limitations of AlphaFold3 in accurately predicting protein interaction interfaces in ion channel complexes.

Due to space limitations, we were unable to present additional applications of AlphaFold3 in the context of Ca_V_ channel complex prediction. For example, post-translational modifications such as phosphorylation and glycosylation are known to play critical roles in regulating Ca_V_ channel gating [73,74]. AlphaFold3 is capable of modeling such modifications. Predicting site-specific post-translational modifications using AlphaFold3 may offer new insights into the structure-function relationship of Ca_V_ channels. However, the extent to which these predictions reflect biological reality remains to be determined through more detailed and systematic studies.

### 5.6 Experimental validation and limitations

While AlphaFold3 provides powerful structural predictions for ion channels such as P2X receptors and Ca_V_ complexes, bridging these computational models with experimental validation remains an essential but nontrivial task. At present, experimental verification is indispensable for assessing the accuracy and physiological relevance of predicted structures.

High-resolution techniques like cryo-electron microscopy (cryo-EM) and X-ray crystallography remain the gold standards for determining protein structures at the atomic level. However, these methods face substantial limitations when applied to highly flexible regions or transient binding interfaces. Notably, AlphaFold3 also tends to perform poorly in these structurally dynamic regions. This convergence of limitations on both the experimental and computational fronts makes accurate modeling and validation of such regions particularly challenging. For ion channels, the transmembrane domain and canonical ligand-binding sites are generally well-structured and relatively stable, making them more amenable to both reliable prediction and structural validation. In contrast, intracellular domains, which are often intrinsically disordered or conformationally heterogeneous to facilitate signaling, remain difficult to resolve experimentally and predict computationally. Further methodological advances will be required to overcome these barriers.

Despite these challenges, functional essays can provide indirect but meaningful evidence for the accuracy of AlphaFold3 predictions. Given that P2X receptors and Ca_V_ channel complex are permeable to Ca^2+^ ions, patch-clamp electrophysiology and calcium imaging are also well-suited for functional characterization, allowing researchers to test hypotheses about channel behavior and ligand/subunit interactions in physiologically relevant conditions. For instance, AlphaFold3 predicts novel interaction interfaces between the Ca_V_ α_1A_ and β_3_ subunits that are absent from current cryo-EM data. These newly predicted binding sites could be tested by targeted point mutations followed by electrophysiological or calcium imaging assays. Such experiments can provide functional validation of AlphaFold3-predicted interfaces, especially in cases where structural data are missing or ambiguous. Moreover, compared to structural biology techniques, these functional assays offer the advantage of assessing ion channel behavior in physiological states. However, it is important to acknowledge the limitations of these indirect methods. Point mutations may cause unintended conformational shifts or destabilize the protein, potentially leading to false-positive or false-negative results. Therefore, complementary approaches, such as testing multiple mutations or combining different assay types, are recommended to increase the robustness of the validation process.

In parallel, MD simulations and molecular docking can provide further insight into conformational changes and ligand interactions, helping to bridge the gap between static structural models and dynamic functional states. Particularly in the context of ion channel gating or allosteric regulation, MD simulations can offer valuable mechanistic insights. Nevertheless, these simulation-based predictions must also be interpreted cautiously and supported by experimental validation.

In summary, AlphaFold3 has greatly expanded the possibilities for structural investigation of ion channels. Yet rigorous validation through structural biology, electrophysiology, and imaging remains essential. These experimental tools will continue to play a critical supervisory role in assessing the accuracy and physiological relevance of AlphaFold3-generated models, particularly in the context of structure-function studies of complex membrane proteins.

## 6 Perspective

While AlphaFold3 marks a substantial breakthrough in protein structure prediction, certain limitations remain. First, the prediction accuracy for unknown proteins, unresolved domains, or those lacking similar structural templates in databases can be relatively low, leading to issues such as chirality errors and atom clashes in large complexes [17]. These drawbacks restrict the broader adoption of AlphaFold in guiding protein design. Nevertheless, recent studies have demonstrated the feasibility of *de novo* ion channel design [75,76], suggesting that ongoing improvements to accuracy and general applicability for structure-unknown proteins are vital to fully harness the potential of computational design [77]. Second, although AlphaFold3 generally predicts accurate backbone architectures for membrane proteins (e.g., ion channels and G-protein coupled receptors, GPCRs), the predicted binding interfaces between ion channels and small molecules (e.g., drugs and ions) often require additional refinement to match experimental accuracy. Furthermore, predictions of interactions between ion channels and biomacromolecules (e.g., proteins, DNA, RNA) remain less reliable, particularly for complexes lacking existing structural templates [78]. Future iterations of AlphaFold or integrated plug-in tools may provide more robust interaction assessments, enabling more efficient virtual screening of channel-interacting partners. Third, AlphaFold predictions usually capture a single conformational state with minimal heterogeneity. In contrast, ion channels operate by transitioning between various states, such as open, closed, and inactivated, which presents a significant challenge for static modeling. Although some emerging methods aim to expand structural ensembles or bias predictions toward specific states [79], their reliability and applicability require further validation. While combining molecular dynamics simulations with AlphaFold can help represent multiple conformations, these methods present a high technical barrier, and the general performance does not always surpass the predictive power of AlphaFold alone [80]. Hence, developing direct strategies to predict distinct functional states of ion channels remains an important goal for future research.

## 7 Summary

**Fig. 8:**
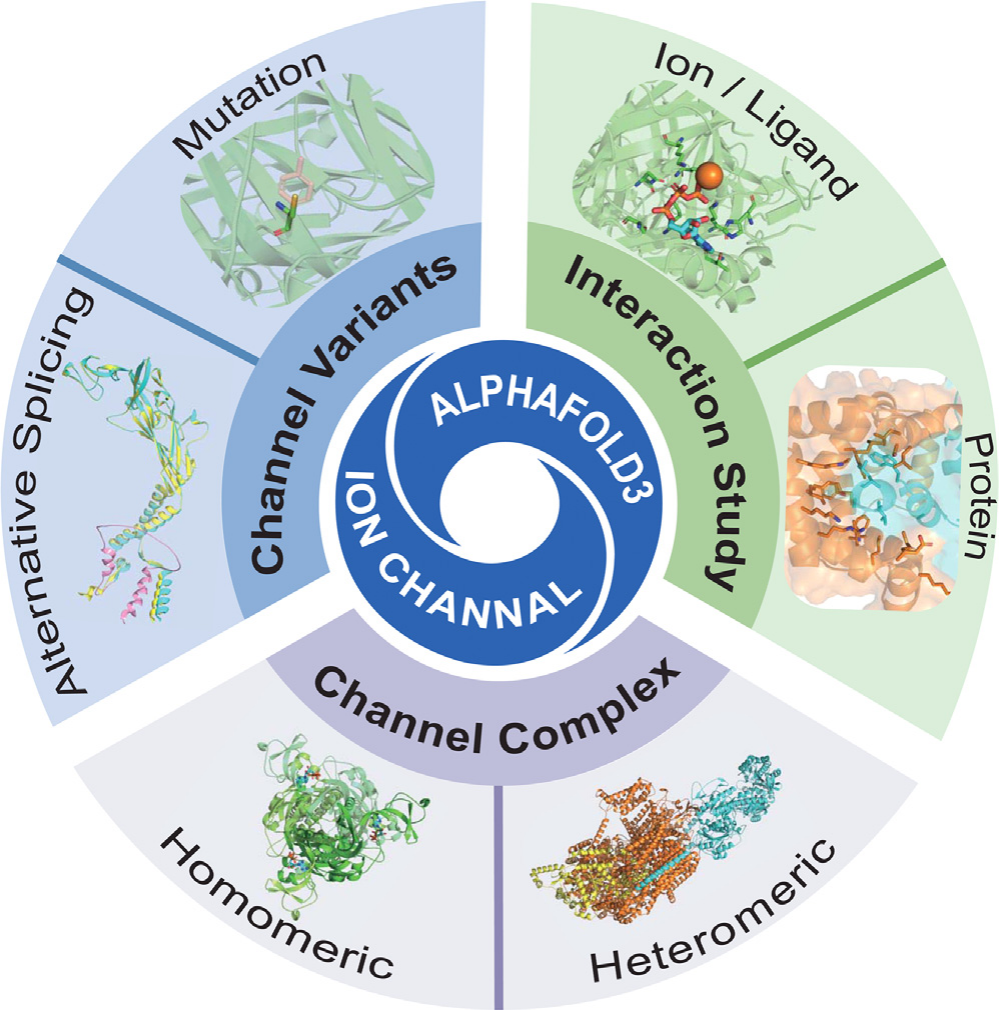
**Overview of AlphaFold3-driven structure-function analyses of ion channels.** In general, AlphaFold3 enables prediction of complex ion channel assemblies, exploration of channel variants, and investigation of channel interactions with ion/ligands or other biomolecules. Figure adapted from AlphaFold Server and Ref. [17], and further processed using PyMOL. Reproduced under the terms of the CC-BY license. Copyright 2024, published by Springer Nature. Reproduced with permission. (For interpretation of the references to colour in this figure legend, the reader is referred to the web version of this article.)

AlphaFold3 is a powerful tool that significantly advances the field of structural and molecular biology. Building on the achievements of its predecessors like AlphaFold2 and AlphaFold-Multimer, it provides enhanced modeling accuracy, particularly for protein complexes, and reduces computational costs. This enables high-precision predictions of protein structures across various research areas, including the study of ion channel structure-function relationships (Fig. 8). Such progress facilitates mechanistic investigations, identification of therapeutic targets, and drug development for ion channels. Although challenges remain, such as the need for experimental validation, structural refinement, and dynamic modeling techniques, the integration of modern computational methods with traditional experimental approaches holds the promise to revolutionize our understanding of ion channels.

## Declaration of generative AI and AI-assisted technologies in the writing process

During the preparation of this work the authors used ChatGPT in order to improve language and readability. After using this tool/service, the authors reviewed and edited the content as needed and take full responsibility for the content of the publication.

## CRediT authorship contribution statement

**Yichen Ke:** Visualization. **Ruijie Gong:** Visualization. **Nan Liu:** Writing – review & editing, Funding acquisition, Supervision. **Yaxiong Yang:** Writing – review & editing, Writing – original draft, Visualization, Supervision, Project administration, Funding acquisition, Conceptualization.

## Declaration of competing interest

The authors declare that they have no conflicts of interest in this work.
